# Diagenesis and clay mineral formation at Gale Crater, Mars

**DOI:** 10.1002/2014JE004757

**Published:** 2015-01-18

**Authors:** J C Bridges, S P Schwenzer, R Leveille, F Westall, R C Wiens, N Mangold, T Bristow, P Edwards, G Berger

**Affiliations:** 1 Space Research Centre, Department of Physics and Astronomy, University of LeicesterLeicester, UK; 2 Department of Physical Sciences, Open UniversityMilton Keynes, UK; 3 Department of Earth and Planetary Science, McGill UniversityMontreal, Quebec, Canada; 4 Centre de Biophysique Moléculaire, CNRSOrléans CEDEX2, France; 5 Space Remote Sensing, Los Alamos National LaboratoryLos Alamos, New Mexico, USA; 6 Laboratoire Planétologie et Géodynamique de Nantes, LPGN/CNRS UMR6112 and Université de NantesNantes, France; 7 Exobiology Branch, NASA Ames Research CenterMoffett Field, California, USA; 8 IRAP (CNRS-Univ. P. Sabatier)Toulouse, France

**Keywords:** Mars, Mars Science Laboratory, clay, Yellowknife Bay, diagenesis

## Abstract

The Mars Science Laboratory rover *Curiosity* found host rocks of basaltic composition and alteration assemblages containing clay minerals at Yellowknife Bay, Gale Crater. On the basis of the observed host rock and alteration minerals, we present results of equilibrium thermochemical modeling of the Sheepbed mudstones of Yellowknife Bay in order to constrain the formation conditions of its secondary mineral assemblage. Building on conclusions from sedimentary observations by the Mars Science Laboratory team, we assume diagenetic, in situ alteration. The modeling shows that the mineral assemblage formed by the reaction of a CO_2_-poor and oxidizing, dilute aqueous solution (Gale Portage Water) in an open system with the Fe-rich basaltic-composition sedimentary rocks at 10–50°C and water/rock ratio (mass of rock reacted with the starting fluid) of 100–1000, pH of ∽7.5–12. Model alteration assemblages predominantly contain phyllosilicates (Fe-smectite, chlorite), the bulk composition of a mixture of which is close to that of saponite inferred from Chemistry and Mineralogy data and to that of saponite observed in the nakhlite Martian meteorites and terrestrial analogues. To match the observed clay mineral chemistry, inhomogeneous dissolution dominated by the amorphous phase and olivine is required. We therefore deduce a dissolving composition of approximately 70% amorphous material, with 20% olivine, and 10% whole rock component.

## 1. Introduction

Gale Crater is thought to have formed near the Noachian-Hesperian boundary with an age of about 3.7 Gyr, and although the exact age of the Gale sediments is not certain, crater counting suggests an ancient age [*Thomson et al*., 2011[. K-Ar dating by the rover Curiosity supports this ancient age by dating a mixture of detrital and authigenic components as found in the Cumberland drill sample to an age of 4.13 ± 0.42 Ga [*Farley et al*., 2014[.

At the Yellowknife Bay locality of Gale Crater, the *Mars Science Laboratory* (MSL) rover *Curiosity* has identified and analyzed, for the first time on Mars, a set of mudstones. The mudstones record a history of deposition within a fluvio-lacustrine environment followed by low temperature, in situ diagenesis [*Grotzinger et al*., 2014; *McLennan et al*., 2014; *Vaniman et al*., 2014[. The composition and mineralogical information preserved in the Gale Crater sediments provide a unique opportunity to determine the nature of the alteration. In particular, we aim to constrain the mineral reactions, Water/rock ratios, pH, and redox conditions associated with the clay- and magnetite-bearing assemblages identified by heMin XRD in the Sheepbed mudstone [*Vaniman et al*., 2014[. We base our model on the sedimentological and mineralogical observations of mudstones and soil observed by the rover Curiosity. The mudstones occur in the Yellowknife Bay area of Gale Crater, about 450 m from the Bradbury landing point. The stratigraphy of the area has been extensively studied from orbit and in the rover images. We give a brief summary from bottom to top of the sequence here, but for details, see *Grotzinger et al*. [2014[, and references therein.

### 1.1 Stratigraphic Overview

**Figure 1:**
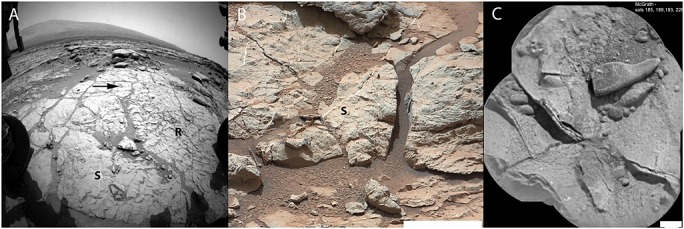
(a) NavCam image of the John Klein outcrop (sol 197). S, sulfate veins; R, raised ridges. The 849 arrow points toward the John Klein drill holes. (b) MastCam image of sulfate veins and nodules 850 within the Sheepbed mudstone outcrop (sol 170). Scale bar of 10 cm. (c) McGrath diagenetic Mg-rich raised ridges within Sheepbed mudstone (sol 234) ChemCam Remote MicroImager. Scale bar of 4 mm.

The 4.5 m thick Yellowknife Bay formation is subdivided into different members with the lowest one, Sheepbed, being an at least 1.5 m thick mudstone, but its lower contact is not visible; its upper contact to the overlying Gillespie member is sharp. The Sheepbed member is a mudstone of overall basaltic chemical composition with ∽15% smectite, ∽50% igneous minerals, and ∽35% X-ray amorphous material [*Grotzinger et al*., 2014[. The observed magnetite is considered to be of authigenic origin [*Grotzinger et al*., 2014[. The unit contains abundant nodules, hollow nodules, voids, raised ridges, and sulfate-filled cracks (Figure 1), all of which are associated with the late stages of the diagenesis [*Grotzinger et al*., 2014; *McLennan et al*., 2014[. Chemistry and Camera (ChemCam) analyses also showed that the raised ridges have a Mg-rich composition (1.2–1.7 times) relative to the surrounding mudstone [*Leveille et al*. 2014[. Key textural observations are that the raised ridges postdate the sedimentary layering and sulfate veins postdate the raised ridges. The notably pure Ca-sulfate composition of the late veins was initially established by ChemCam (Laser Induced Breakdown Spectroscopy) and was confirmed by Alpha Proton X-ray Spectrometer (APXS) [*McLennan et al*., 2014[. Both drilled samples—named John Klein and Cumberland—are within the Sheepbed member [*Vaniman et al*., 2014[.

The Sheepbed mudstone has a sharp contact with the overlying 3 m thick succession of the Gillespie and Glenelg members, which contain fluvial sediments [e.g., *Grotzinger et al*., 2014[, with a lower abundance of sulfate veining than Sheepbed. The Yellowknife Bay formation underlies the Hottah Facies conglomerates found across the Peace Vallis alluvial fan [*Williams et al*., 2013[. The upper and youngest sediments on the area are unconsolidated, windblown soils, which were investigated with the rover instruments at the Rocknest site [*Bish et al*., 2013; *Morris et al*., 2014[.

### 1.2 John Klein and Cumberland Drill Results and Implications for Environmental Conditions

Two drilled samples of the mudstone, at locations named John_Klein and Cumberland, took place between Martian solar days (sols) 180 and 292 of the mission and allowed analysis of material beneath the uppermost, reddish oxidized dust coating. The samples were analyzed in the CheMin instrument by X-ray diffraction [*Vaniman et al*., 2014[ (Table 1) and by pyrolysis with the Sample Analysis at Mars (SAM) gas chromatograph–mass spectrometer [*Ming et al*., 2014[ in order to obtain the mineral identities and evolved gas compositions. Both CheMin analyses revealed a saponite in the Sheepbed mudstone, and geochemical observations [*McLennan et al*., 2014[ suggest only minor chemical alteration of the sediment source region before deposition. The mineralogical and sedimentological observations suggested that Yellowknife Bay has been a habitable environment, with a neutral to alkaline pH and relatively low temperatures of diagenesis [*Grotzinger et al*., 2014[. Furthermore, *McLennan et al*. [2014[ suggested on the basis of major element discriminant diagrams and Chemical Index of Alteration criteria from *Nesbitt* [2003[ that the Yellowknife Bay formation had very little evidence of chemical mobility associated with the alteration. They conclude that this indicated arid, possibly cold, palaeoclimates with rapid erosion and deposition and low water/rock ratios during diagenesis.

**Table 1:** CheMin Mineral Abundances as Reported by *Bish et al*. [2013[ and *Vaniman et al*. [2014[a

Mineral	Portage Soil	John Klein	Cumberland
Plagioclase	40.8	44.8	41
Fe-forsterite	22.4	5.7	1.9
Augite	14.6	7.6	9
Pigeonite	13.8	11.3	16
Orthopyroxene		6.1	9
Magnetite	2.1	7.6	9
Anhydrite	1.5	5.3	
Bassanite		2.1	1.2
Quartz	1.4	0.9^*^	0.2^*^
Sanidine	1.3^*^	2.4	3.5
Hematite	1.1^*^	1.2^*^	1.3
Ilmenite	0.9^*^		1.2^*^
Akaganeite		2.3	3
Halite		0.3^*^	0.3^*^
Pyrite		0.6^*^	
Pyrrhotite		2.0	1.9
Amorphous	27	28	31
Clay		22	18

a Data from *Bish et al*. [2013[ and *Vaniman et al*. [2014[. Asterisks mean at or near detection limits. Note that the crystalline components (minus clay and amorphous) are normalized to 100%.

### 1.3 Rocknest Soil and APXS Rock Analysis

CheMin and APXS analyses of the Portage soil were carried out between sols 55 and 102 at the Rocknest locality. This provides a mineralogical control on the country rock in the Gale Crater region [*Bish et al*., 2013, and references therein[. Two of the major phases identified by CheMin were forsteritic olivine and an amorphous component together with plagioclase, augite, pigeonite, and minor minerals but no clay [*Bish et al*., 2013[ (see Table 1). The amorphous component is interpreted as being similar to an amorphous component found in Hawaiian basaltic soils [*Bish et al*., 2013[.

Chemically, the APXS analyses of other Gale Crater rocks have established the presence of a range of compositions. These include Fe-rich basaltic sediment as shown by the in situ analyses at Yellowknife Bay and the Portage soil analysis [*Schmidt et al*., 2014; *McLennan et al*., 2014[. A large range in alkali compositions has been seen in other samples, including a K-rich alkaline basaltic composition shown by the Jake_Matijevic sample [*Stolper et al*., 2013; *Schmidt et al*., 2014[. Rock samples found within the Rocknest (sols 55–102) and Bathurst_Inlet (sol 54) localities are probable basaltic sediments with alkaline contents intermediate between those of Jake_Matijevic and Portage soil or the Sheepbed mudstones [*Schmidt et al*., 2014[.

By using the sedimentological constraints together with ChemCam and APXS major element analyses of representative basaltic and alkaline compositions of the Gale Crater rocks and soil, and the CheMin and SAM results during the first 300 sols, we establish an equilibrium thermochemical model for the subsurface mineral reactions in the Yellowknife Bay sediments of Gale Crater. This model envisages reaction of a pore water (Gale Portage Water (GPW), see Methods) with the enclosing detrital sediment. In our model, we primarily study the production of clay through the inhomogeneous alteration of a Rocknest-type host rock, within which olivine and amorphous material are the predominant alteration phases, because both of which are relatively reactive compared to other phases. We also consider other host rock end-members (see Methods). There is clear evidence from terrestrial analogue environments such as altered Icelandic basalts and tuffs that olivine and glassy material are the most reactive phases [e.g., *Bishop et al*., 2002[. We use the thermochemical model to provide a way of understanding of the secondary minerals of Gale Crater that is complementary to the field observations made by the *Curiosity* team. Starting with unaltered rocks and soils found in the area, we aim to calculate a realistic mixture of dissolving minerals within those rocks and soils that reacted to form the secondary, clay-bearing assemblage during diagenesis. This will also help to decide whether some of the phases are detrital or authigenic or a mixture of both.

## 2. Model Methods and Assumptions

For the thermochemical modeling, we use the program CHIM-XPT (previously CHILLER) [*Reed and Spycher*, 2006; *Reed et al*., 2010[, which is a program for computing multicomponent, heterogeneous chemical equilibria. This means that every calculation step calculates equilibrium between the starting fluid and the dissolved rock. Thus, each step can be treated and interpreted independently from the direction from which it was reached, and trends in water/rock (W/R) ratio can be read in both directions, because equilibrium is independently calculated for each step. Step size may vary depending on the requirements of the task, and the calculation is largely independent of the amount of water, since a weight ratio is used and the base unit for the calculation is moles. For convenience, 1 kg (or 55.5 mol) of water is generally the basis for the calculation. The method used here is a batch calculation where precipitates are not fractionated from the system. For details of the code, database and input files, we refer to the handbook for CHIM-XPT [*Reed et al*., 2010[ for background on the reaction pathway models, in general, relevant to our paper's methodology, especially titration modeling, see example *Kühn* [2004, chapter 3[ and for a discussion on databases and the mathematical-theoretical background [see, e.g., *Ganguly*, 2008 and *Oelkers and Schott*, 2009, especially chapters 1–3[. CHIM-XPT has been extensively used in terrestrial basaltic environments [e.g., *Reed*, 1982; *Reed*, 1983[ and for Martian compositions [*DeBraal et al*., 1993; *De Caritat et al*., 1993; *Schwenzer and Kring*, 2009; *Bridges and Schwenzer*, 2012; *Schwenzer et al*., 2012a, 2012b; *Schwenzer and Kring*, 2013; *Filiberto and Schwenzer*, 2013[. The program requires choices on starting fluid, host rock elemental composition, temperature, and pressure.

For host rock compositions in our modeling, we used a variety of rocks observed by Curiosity (Table 2 and section 2.2) and, in addition, selective mineral reactions in a Portage-soil type host rock with the amorphous component and olivine. Note that we start with a rock that has 22.4% olivine and no clay minerals (Portage soil; Table 1) and model the alteration minerals as they are found in the mudstones (John Klein and Cumberland drill samples; Table 1), which contain much less olivine (6 and 2%, respectively) and 20 ± 2% phyllosilicates [*Vaniman et al*., 2014[ (Table 1). Temperature was set to 10°C and pressure to 1 bar for the models presented here, following the diagenetic scenario suggested by the sedimentological observations [*Grotzinger et al*., 2014[.

**Table 2:** Compositions of Starting Rock, Soil, and Single Phase Compositions^a^

					Amorphous		
Wt %	Jake_M	Ekwir Brushed	Portage	Olivine	Portage	John Klein	Cumberland
SiO_2_	51.85	46.07	44.88	36.3	41.78	41.01	41.73
TiO_2_	0.51	0.90	1.25		2.25	2.07	1.66
Al_2_O_3_	16.51	8.43	9.87	0.06	6.95	6.26	6.00
Cr_2_O_3_	0.03	0.37	0.51	0.02	1.17	0.90	0.91
FeO	6.43	13.27	12.96	33.2	7.78	0.35	21.45
Fe_2_O_3_	1.07	1.64	2.23		15.74	20.70	2.78
MnO	0.14	0.21	0.43	0.63	0.98	0.55	0.54
MgO	3.71	9.83	9.09	29.7	5.72	7.81	8.35
CaO	6.23	6.00	7.62	0.25	6.37	8.97	6.80
Na_2_O	6.47	1.34	2.22		2.96	3.36	2.49
NaCl	1.48	2.99	1.19		1.77	1.44	3.17
K_2_O	2.27	0.63	0.51		0.95	1.00	0.79
P_2_O_5_	0.51	1.10	0.98		2.24	1.98	2.02
FeS	2.76	7.23	6.26		3.35	3.60	1.30

a The 10% of the total Fe (molar) is taken as Fe^3+^, and all S is recalculated as FeS or—for high S concentrations in the soils, FeS_2_, respectively. Whole rocks (Jake_M, Ekwir_brushed and Portage are as measured by APXS [*Gellert et al*., 2013[, olivine from *Vaniman et al*. [2014[, and amorphous compositions from *Morris et al*. [2014[ for Portage, John Klein, and Cumberland. Note that the data have been recalculated to represent model components; therefore, S species are represented with FeS and FeS_2_, and Cl is represented as NaCl. Redox conditions have been set to 10% Fe (molar) as Fe^3+^, except for olivine, which is 0% and Portage amorphous, which is 42%.

Results of calculated equilibrium mineral assemblages are presented in diagrams of mineral abundance versus W/R ratio (mass of rock *reacted* with the starting fluid). The plotted W/R ratio is thus a progress variable with very limited rock dissolution at the high W/R end and increased rock dissolution at the low W/R end. Note that W/R end represents the amount of rock reacted with the fluid not the total amount of rock present in a given volume of rock on Mars. Original magmatic minerals are observed in the mudstones [*Vaniman et al*., 2014[ (Table 1), which means that the alteration of the rock is incomplete and unreacted material remains. Therefore, for comparison to the overall water to rock ratio as addressed by bulk rock compositional trends [*McLennan et al*., 2014[, assumptions about the amount of reacted rock per total unit volume of rock in Gale have to be made. In other words, high W/R ratios might be indicative of systems, where the water interacts with a limited surface area, and therefore, only a small mass of rock is dissolved in a large mass of water. This occurs for example in a fracture, or on a rock surface exposed to regular precipitation. Low W/R ratio might occur where large rock surfaces react with a volume of water that is stagnant and not exchanged, e.g., in a porous sediment, although our results in succeeding sections suggest an open system with inflowing water, rather than a stagnant situation. The exact amount of precipitate caused by this dissolution is dependent on the species remaining in solution and on the details of minerals precipitated, specifically on structurally bound water or incorporated CO_2_. The amount of precipitation increases from a few milligram at high W/R to about 1 g at W/R of 1000 and on the order of 10 g at W/R of 100. We model between W/R of 1 and 100000 but only show 10 to 10000 for most of the runs. Higher W/R is unlikely within a sediment, but the lowest W/R would also produce phases with less H_2_O than phyllosilicates. W/R therefore describes the environment (freshwater inflow at high W/R in contrast to stagnant fluids with no fresh inflow at the low W/R), but at the same time reaction progress, because in a stagnant situation, more host rock will react over time, especially at low temperatures, where reactions are slow.

### 2.1 The Starting Fluid Composition: Gale Portage Water

In order to model a realistic starting fluid representative of water associated with diagenesis in the Yellowknife Bay sediments, we start with adapted water (AW). This is the fluid used in our previous Mars studies [see *Schwenzer and Kring*, 2009[. It is a dilute aqueous solution with species concentrations based on warm fluids venting from a terrestrial basaltic environment—the Deccan Traps [*Minissale et al*., 2000[. The Deccan Traps were chosen, because of the seawater-free nature of this environment. The fluid was then adjusted for Martian basaltic compositions by taking the Ca-concentration of the terrestrial fluid and adjusting the Mg and Fe contents using the Ca/Fe and Ca/Mg ratios observed in Martian rock (shergottite LEW 88516). The solution is initially oxidizing (all S species as SO_4_^2−^). CHIM-XPT can be controlled either by the set of O_2_-H_2_O-SO_4_-H^+^ or expressed in terms of HS-SO_4_-H_2_O-H^+^. During the reactions, the SO_4_^2−^/HS^−^ pair controls redox in the fluid [*Reed et al*., 2010[, and a set of 112 different ionic species are typically used to represent the fluid chemistry in each calculation step. Table 3 is a summary representation of the element concentrations. The redox of the system throughout the run is dependent on the Fe^2+^/Fe^3+^ ratio of the host rock or soil (see section 2.3.2). Sulfur concentration of the fluid was taken as found in the Deccan Trap fluids [*Minissale et al*., 2000[, and chlorine was used as the charge balance ion. From this, a dilute version was calculated by dividing all species concentrations by 10,000. This reduces the influence of introduced species in our model.

**Table 3:** Fluid Compositions Used in the Modeling[Table-fn tf3-1]

	AW	GPW	GPW 185 mbar
Cl^−^	0.587E-1	5.76E-3	5.76E-3
SO_4_^2−^	0.285E-2	3.97E-3	3.97E-3
HCO_3_^−^	0.168E-4	1.68E-4	0.62E-2
SiO_2_	-	3.49E-5	3.49E-5
Ca^2+^	0.250E-2	1.41E-5	1.41E-5
Mg^2+^	0.205E-1	1.27E-8	1.27E-8
Fe^2+^	0.919E-2	-	-
K^+^	-	5.02E-4	5.02E-4
Na^+^	-	9.20E-3	9.20E-3
Mn^2+^		4.36E-8	4.36E-8

Adapted water (AW) is a fluid calculated from Deccan Trap fluids [*Minissale et al*., 2000[ and adapted to Martian Fe/Ca/Mg ratios. For details, see text and *Schwenzer and Kring* [2009[. Gale Portage Water (GPW) is a fluid deduced from an initial reaction of diluted AW (×10,000) with rocks at Gale Crater (see text). Note that this table summarizes element concentrations, but those are represented typically by a set of 112 ionic species during runs. Only values above 10^−10^ were taken into account for GPW. Units are shown in moles.

**Figure 2:**
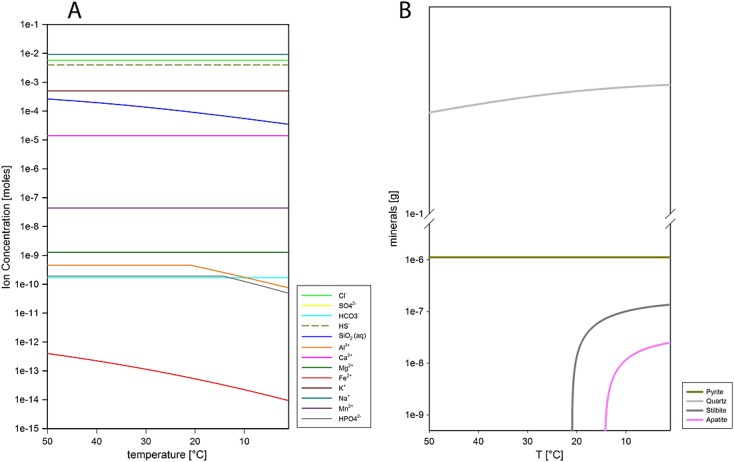
Portage soil has been reacted with dilute Adapted Water AW at 50°C. The fluid was extracted from the original reaction at W/R (ratio of reacted rock with incoming fluid) of 100 and subsequently cooled to form Gale Portage water GPW, which we use in our model runs for the Yellowknife Bay diagenesis assemblage. (a) Plot of temperature (*T* in °C) versus ion concentration in 1 kg of water (in mol) of the fluid in equilibrium with the precipitate at different temperatures. Cooling causes precipitation—most noticeably of SiO_2_, Fe, Al and S. (b) Minerals precipitated upon cooling. Main precipitates are quartz (or another SiO_2_ phase, depending on reaction kinetics), pyrite, stilbite, and apatite.

Next, solid of Portage soil composition (Table 1) was titrated into this fluid at 50°C and 1 bar to account for a reaction of buried sediment (potentially at a higher geothermal gradient post impact) with the country rock. Portage soil from the Rocknest sand shadow is taken to be representative of average crustal compositions in the vicinity of Gale Crater. The resulting fluid composition at W/R of 100 was separated from the clay precipitate and cooled to 1°C (Figure 2), during which it produced a quartz (or amorphous SiO_2_, depending on kinetics) dominated precipitate (Figure 2). This is a common feature of cooling alteration fluids, and there is evidence for silica-rich deposits on Mars, probably forming under a variety of temperatures and other conditions [e.g., *McAdam et al*., 2008; *Squyres et al*., 2008[. The Gale fluid was again separated from the precipitate, and the ions left in the fluid were considered to be GPW. CO_2_—as a proxy for C-bearing species—is added as 1.68E-4 mol HCO_3_^−^, a concentration that precludes carbonate formation, consistent with MSL results, and used in our previous work [e.g., *Schwenzer and Kring*, 2009[. All S species in GPW are summarized as SO_4_^−^. Species with concentrations below 10^−10^ mol were not considered in this starting fluid composition.

### 2.2 Starting Rock Compositions and Model Conditions

Reactions using GPW were calculated with different Gale rocks (Table 2) in order to explain the phyllosilicates observed in the John Klein and Cumberland drill samples. We use Jake_Matijevic as an alkaline end-member, Ekwir_brushed as an average, relatively dust-free basaltic composition, and Portage as the least altered basaltic soil end-member. We focus on Portage soil, because rover investigations returned the chemistry and mineralogy of the sample [*Morris et al*., 2014; *Vaniman et al*., 2014[. Portage soil also gives a representative regional composition of unaltered basaltic host rocks, because most rocks (including Portage soil and the clay-bearing lake bed mudstones) are chemically similar to typical upper Martian crust [*Grotzinger et al*., 2014[.

For other calculations, we used the varying proportions of individual components, e.g., olivine and amorphous component. In total, over 100 runs with varying composition, temperature, and redox conditions were performed. APXS analyses were used for rock and soil compositions [*Gellert et al*., 2013; *Stolper et al*., 2013; *Schmidt et al*., 2014[, and following a set of runs (section 3.1) to explore the effect of varying the rock's redox state on the alteration mineral assemblage, we took 10% of the total Fe (molar) to be Fe^3+^. We also took 10% Fe^3+^ for the John Klein and Cumberland amorphous compositions, but for Portage amorphous, we used the existing estimate by *Morris et al*. [2014[, which is 42% Fe^3+^ (Table 2), thus giving us a range of potential Fe^3+^ contents associated with the amorphous component. SO_3_ from the APXS data was recalculated as FeS or FeS_2_, subtracting the equivalent amount of Fe from FeO. Chlorine was recalculated as NaCl, and the equivalent amount of Na was subtracted from Na_2_O. We tested three different temperatures, 10°C, 50°C, and 150°C, and note that at the lowest temperature, some minerals known to form only at higher temperatures in nature (e.g., pyroxenes and amphiboles) were excluded from forming during the runs.

The bulk rock models provide insights into the expected alteration mineralogy associated with the general chemistry of the rocks encountered at Yellowknife Bay. However, mineral dissolution is inhomogeneous and highly dependent on temperature and fluid chemistry [e.g., *Zolotov and Mironenko*, 2007; *Hausrath et al*., 2008; *McAdam et al*., 2008; *Gudbrandsson et al*., 2011[. Thus, we also calculated reactions with different mineral mixtures, taking a similar approach to that in our previous nakhlite Martian meteorite models [*Bridges and Schwenzer*, 2012[. Some information about mineral dissolution can be deduced from the observations of the differences in the concentrations of olivine, magnetite, and amorphous component between samples [*Vaniman et al*., 2014[ (Table 1), although we do not directly consider mineral dissolution rates in this paper. The Sheepbed mudstone contains 22 wt % (John Klein drill hole) to 18 wt % (Cumberland drill hole) saponite in a basaltic, Fe-sulfide-bearing mineral assemblage, with possible traces of quartz; it also contains substantial amorphous material [*Vaniman et al*., 2014[. The Portage soil contained no crystalline phyllosilicates [*Bish et al*., 2013[. We use this information to deduce a variety of starting mineral mixes, ranging from pure olivine and pure amorphous component to mixtures of olivine, amorphous component, and host rock chemistry.

We use the following sheet silicates in our CHIM-XPT database: talc (Mg, Mg-Al, Fe end-members); pyrophyllite; from the chlorite group clinochlore, daphnite, Mn-chlorite, Al-free chlorite; from the kaolinite group kaolinite, illite; from the smectite group montmorillonite (Na, K, Mg, Ca end-members); beidellite (H, Na, K, Mg, Ca end-members), nontronite (H, Na, K, Mg, Ca, Fe end-members); and serpentine (antigorite, chrysotile, greenalite). We note that there is no other kaolinite group mineral other than kaolinite, e.g., vermiculite, saponite, and hectorite are not in our database. In the interpretation of our models, nontronite serves as the Fe^3+^ clay and the daphnite end-members of chlorite as the Fe^2+^ clay. In our plots of mineral abundance versus W/R, we plot the combined end-members of chlorite. Phases that are not known to form at low temperatures were excluded from the runs, these include garnet, amphiboles, and pyroxenes, as well as high-*T* mica.

As outlined above, we use a set of assumptions about the redox conditions of the starting fluid and the redox conditions in the dissolving rock. Bearing in mind the uncertainty of the Fe^2+^/Fe^3+^ in those assumptions, and the fact that the thermochemical database is necessarily limited relative to the full large range of possible natural mineral assemblages, we have modeled a best chemical match for the observed clays. Thus, in our runs, we take a nontronite + chlorite assemblage as a chemical analogue to the clay identified by *Vaniman et al*. [2014[ with XRD data. These phases are relevant to the low temperature, diagenetic type of environment we are considering [e.g., *de Caritat et al*., 1993[, although they might, however, form as mixed layer clays [*Ryan and Reynolds*, 1997[. All phyllosilicates in the model are added to derive the clay chemistry with a weighted average composition. We also compare the calculated compositions to ferric phyllosilicates observed in the nakhlite Martian meteorites [*Changela and Bridges*, 2010; *Hicks et al*., 2014[ and a terrestrial, griffithite analogue [*Treiman et al*., 2014[.

Carbonates were not detected by CheMin [*Vaniman et al*., 2014[, although SAM analysis of the borehole fines did suggest the potential presence of a carbonate [*Ming et al*., 2014[. By taking Martian meteorite ALH84001 carbonate as an average of the top 1 km of Martian crust, *Bridges et al*. [2001[, calculated an equivalent CO_2_ partial pressure of 185 mbar and thus a possible atmospheric pressure associated with ancient Mars. Therefore, to test the influence of CO_2_ dissolved in the incoming fluid, Portage soil was exposed to GPW fluid equilibrated with 185 mbar CO_2_ (0.62 × 10^−2^ mol CO_2_/kg H_2_O; Tables 2 and 3) for some of our runs. Because the most likely formation process of the clays is diagenetic [*Vaniman et al*., 2014; *Bristow et al*., 2014[, we assume that the system is closed to the atmosphere; i.e., no CO_2_ replenishment was possible to balance any carbonate precipitation.

## 3. Results

### 3.1 Models With Varying Fe^3+^ Content

**Figure 3:**
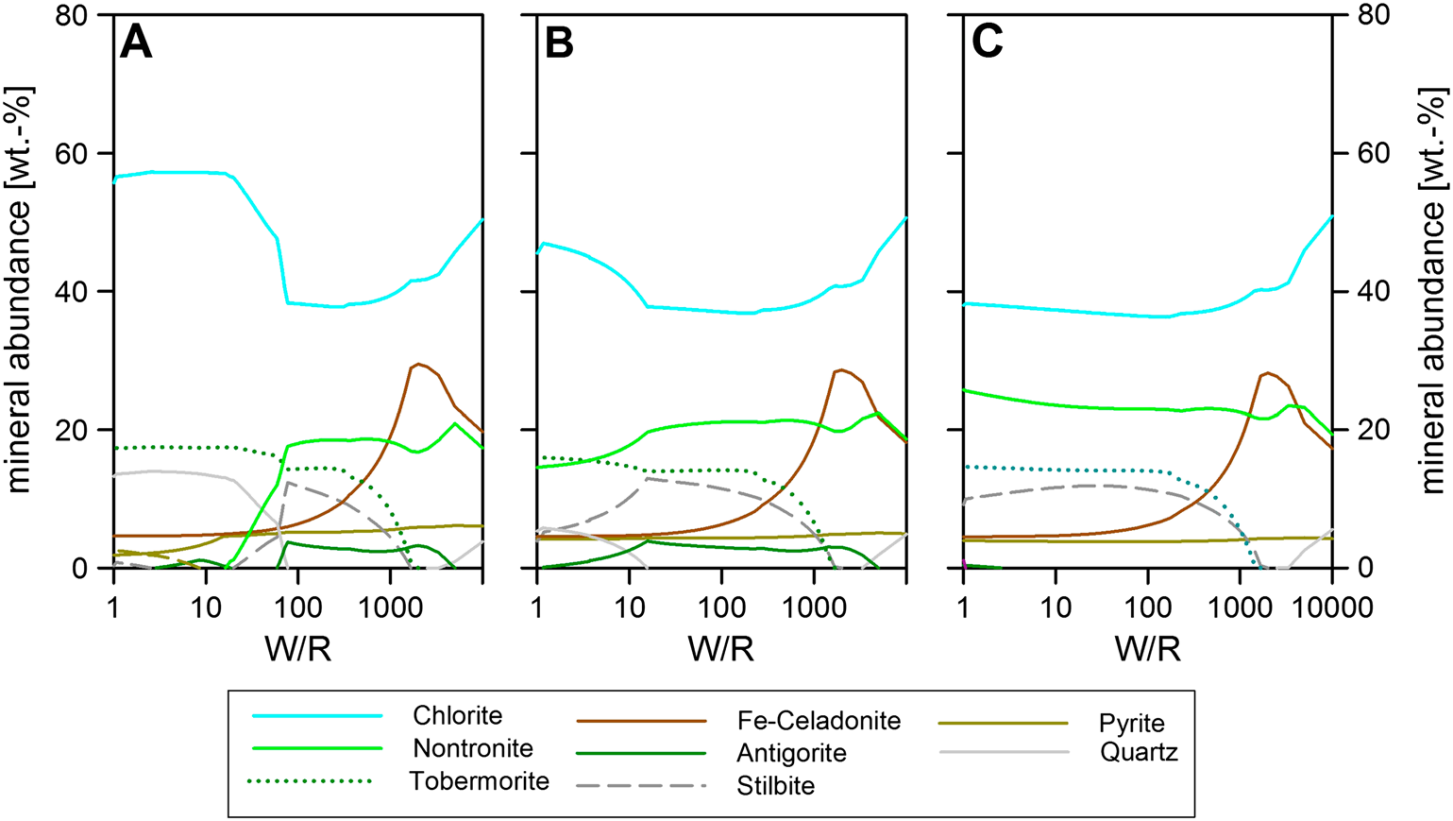
Portage soil reacted with GPW: (a) 10°C and 10% (molar) of the Fe is Fe^3+^, (b) 50% Fe as Fe^3+^, (c) and 74.5% of Fe as Fe^3+^, which is equivalent to all Fe in silicates being Fe^3+^. Trace phases (below 3%) are not plotted and include apatite for all models. The results of these runs have led us to use the 10% Fe^3+^ composition in the rest of the models for the whole rock components of our runs.

**Figure 4:**
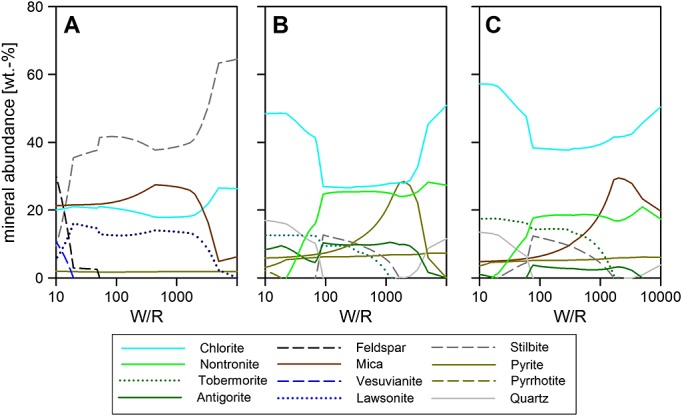
Whole rock compositions reacted with Gale Portage Water (GPW). Reactions at 10°C. (a) Jake_M, (b) Ekwir brushed, and (c) Portage Soil. Minerals not plotted (below 3% abundance): apatite and alabandite. W/R is the ratio of incoming fluid with reacted rock.

**Figure 5:**
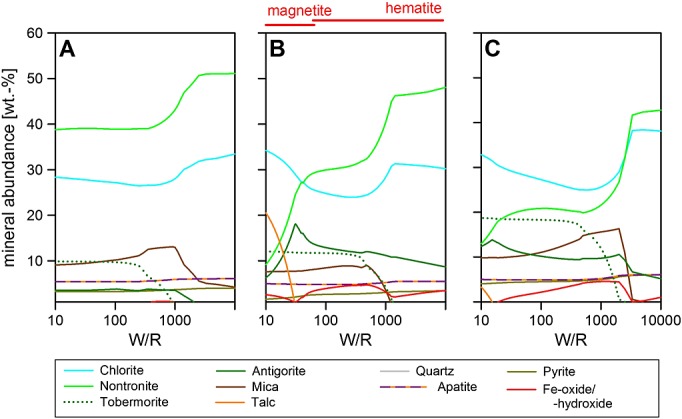
Three different amorphous components (a) Portage [*Morris et al*., 2014[, (b) Cumberland, and (c) John Klein, reacted with GPW at 10°C. For compositional details, see Table 2. The results of these runs are also plotted on the clay ternary (Figure 8). The Fe oxide in Figure 5c is goethite. W/R is the ratio of incoming fluid with reacted rock.

**Figure 6:**
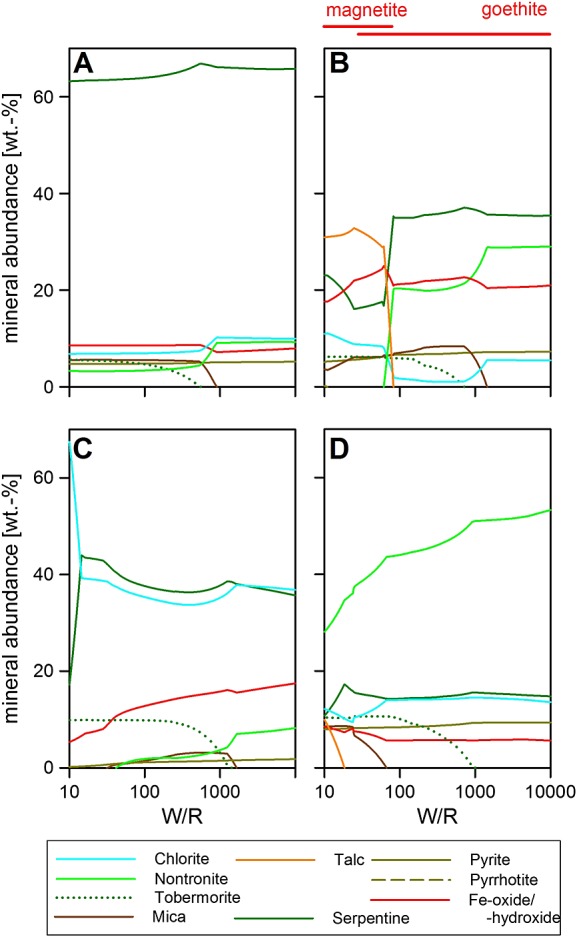
Examples of different mixtures of components found in Portage (Tables 1 and 2) reacted with GPW at 10°C. (a) 50% forsterite and 50% Portage amorphous; (b) 50% olivine (Mg_1.24_Fe_0.76_SiO_4_) and 50% Portage amorphous; (c) 70% olivine, 15% augite, and 15% plagioclase; and (d) 70% Portage amorphous, 20% olivine, and 10% whole rock. The Fe oxide in Figure 6a is a trace of goethite, in Figure 6c, there is a switch from goethite to magnetite at W/R = 15. In Figure 6d, the oxide is forced to be magnetite, but no major change is observed in the clay composition compared to an unforced run. The bulk phyllosilicate results are also plotted on the clay ternary Figure 8. W/R is the ratio of incoming fluid with reacted rock.

The Fe^2+^/Fe^3+^ ratios in the Gale Crater host rocks are not known precisely. Previous studies of Martian water-rock interaction have taken 10% Fe as Fe^3+^ because of the assumed redox state in the basaltic shergottites, for details see *Schwenzer and Kring* [2009, 2013[ and *Filiberto and Schwenzer* [2013[. In order to test the validity of the 10% figure for the whole rock samples studied here, we studied the influence of varying host rock redox conditions with 10–75% Fe as Fe^3+^ (Figure 3). Because the soil is the most likely host rock to be influenced by other factors, such as evaporite deposition or atmosphere-soil interactions, the test was done on Portage soil. Figure 3a shows the same model as Figure 4c for the ease of comparison. Figures 3b and 3c demonstrate that with increasing Fe^3+^ content in the host rock, the W/R range at which nontronite is the second most abundant phase extends to much lower W/R, because nontronite is the Fe^3+^ phase in the precipitate. The amount of stilbite increases at low W/R with increasing Fe^3+^ content, which is likely to be the result of changes in the solubility of Ca and Si. The overall result of a chlorite-dominated assemblage does not change between the different Fe^3+^/Fe_tot_ ratio runs, and we therefore select the 10% figure for the whole rock component because it is consistent with both our results and previous work on other Martian systems. However, the bulk Fe^3+^/Fe_tot_ ratio in our runs (Figures 5 and 6) varies from 0 to 45% as the proportion of the components, whole rock, amorphous, olivine, whole rock, and plagioclase, is varied.

### 3.2 Models With Whole Rock Compositions

Starting with Jake_M as the starting material at 10°C, with 10% of the Fe as Fe^3+^, the precipitates are dominated by zeolite (stilbite) over almost all of the modeled W/R range; there is over 40% between W/R of 10,000 and ∽20 (Figure 4a). The most abundant phases above W/R of ∽20 are sheet silicates—chlorite or Fe-celadonite (Figure 4a), which are replaced by secondary Na-feldspar (and vesuvianite) at very low W/R (∽13). Lawsonite contributes with more than 10% abundance between W/R of 2500 and 14.

In contrast to Jake_M, Ekwir_brushed (Figure 4b), precipitates are dominated by chlorite and nontronite at all W/R. Chlorite minerals have a total abundance of at least 25–60% and are the most abundant phase throughout. Nontronite abundance drops from 28% at W/R of 5000 to 10% at W/R of 40 and is not part of the precipitated assemblage below W/R of 20. Two other sheet silicates are present: Fe-celadonite, which reaches its highest abundance (28%) at W/R of 2000, after which it steadily declines with decreasing W/R, reaching 10% at W/R of 270 and remaining constant at 6% for W/R of 40 and below. Serpentine formation occurs at W/R of 5000 and quickly reaches 10% between W/R of 2500 and 90, after which a slight dip in serpentine abundance occurs, before another plateau at an abundance of ∽7% is reached. Stilbite is observed between W/R 1500 and 70, and SiO_2_-phase occurs when stilbite does not form. Tobermorite takes up Ca not contained in the sheet silicates (below W/R 1000).

Portage soil (Figure 4c) alteration phases are very similar to Ekwir_brushed with chlorite dominating the assemblage (above 40% at all W/R). Nontronite remains the second most abundant phase at intermediate W/R. Like the Ekwir runs, pyrite is present over a wide range of W/R, at abundances around 5% at high to intermediate W/R and below 2% at lower W/R (<20). Because of the higher CaO and lower SiO_2_ contents of the host rock (Table 2), tobermorite is present at higher abundances in Portage than in Ekwir alteration products, while the SiO_2_-phase is less abundant in Portage-derived alteration assemblages.

From this, it becomes clear that the basaltic compositions of Portage soil and Ekwir_brushed, when reacted with GPW at 10–50°C (50°C not shown here, because of the similarity to the 10°C runs), and at W/R mass of rock reacted with the incoming fluid of 100–1000, produce calculated mineral assemblages that most closely match the saponite-, sulfide-, and Fe oxide-bearing assemblages identified in the Sheepbed mudstone by CheMin [*Vaniman et al*., 2014[. We note that the calculated clay is a chemical, not a mineralogical analogue, and that (as outlined in more detail below) the alkaline composition Jake_M is not a likely source for the secondary mineralogy at Yellowknife Bay as neither kaolinite nor serpentine and zeolites are present in the secondary assemblage. High-temperature, hydrothermal mineral assemblages ≥150°C, derived from either basaltic or alkaline rocks, in the Gale Crater rim, and transported as detrital grains into the Sheepbed mudstone, are also unlikely to be major constituents in the Sheepbed mudstones, as high temperature models with the same compositions that show that this material would not only include kaolinite but also amphiboles, which have not been identified.

### 3.3 Mineral and Amorphous Component Reactions

The CheMin analysis on Rocknest soil and Cumberland and John Klein drill fines returned three different amorphous compositions in the samples [*Morris et al*., 2014; *Vaniman et al*., 2014[ (Table 2). While in Portage, the amorphous component is interpreted as glassy component similar to what is found in terrestrial basaltic sediments [*Bish et al*., 2013[; in the mudstones, they could be a mix of this glassy component and altered glass. We have modeled the reaction of all three amorphous components to hydrous alteration products. We note that very low W/R conditions could involve alteration of the glassy amorphous component with very little H_2_O. In contrast, in the nakhlite meteorites, the amorphous component is the last product of alteration and has a similar composition to the crystalline saponite phase, and crystallization of the amorphous gel is assumed to have been inhibited by kinetic effects [*Changela and Bridges*, 2010; *Hicks et al*., 2014[. The exact nature of the amorphous component and the succession of events cannot be studied with Curiosity instrumentation. We therefore take it as part of the starting material only in our model calculations.

Figure 5 shows that all three amorphous compositions from Gale Crater could form a nontronite and chlorite-dominated assemblage. However, because of the higher predicted Fe^3+^ in the Portage amorphous component [see also *Morris et al*., 2014[ (Table 2), nontronite dominates across all W/R for that sample. For Cumberland and John Klein, chlorite becomes more dominant at lower W/R, ∽50 and 5000, respectively. For Portage, all other minerals stay below 10 wt % at all W/R, but the higher Mg abundance in Cumberland and John Klein results in a more variable alteration assemblage, especially at low W/R, e.g., forming more serpentine and talc.

To take inhomogeneous dissolution (see section 2.2) into account, we have modeled the reaction of a variety of mixtures leading from that observation: the minerals and mineral mixtures are ranging from pure olivine as observed in Gale (Mg_1.24_Fe_0.76_SiO_4_), pure forsterite (Mg_2_SiO_4_), olivine + host rock, olivine + amorphous, forsterite or fayalite + amorphous, and mixtures of all three components. We show (a) 50% forsterite and 50% Portage amorphous, Fe^3+^/Fe_tot_ = 45%; (b) 50% forsterite and 50% Portage amorphous, Fe^3+^/Fe_tot_ = 45%; (c) 70% olivine, 15% augite, 15% plagioclase, and Fe^3+^/Fe_tot_ = 0%; and (d) 70% Portage amorphous, 20% olivine, and 10% whole rock Fe^3+^/Fe_tot_ = 40% in Figure 6. Note that we show W/R of 10 (referred to as “low”) to 10,000 (referred to as high), because this is the range of W/R most realistic for the diagenetic, incomplete reactions at low *T*. The bulk W/R of the rock unit is lower than the W/R of the models because of the presence of unreacted minerals in the mudstone, see also section 2 for more details. Pure forsterite runs produce an Al-free brucite dominated assemblage, which does not match the Al-bearing nature of the observed phyllosilicates. In contrast, other olivine-rich runs produce the expected serpentine-Fe oxide-SiO_2_ assemblage. A mixture of forsterite and amorphous component produces precipitates dominated by serpentine and ∽10% of nontronite, chlorite, and Fe oxide each (Figure 6a), while a mixture of amorphous component and olivine (1:1; Figure 6b) returns a serpentine-dominated assemblage above W/R 65% and talc below that. Nontronite is the second most abundant phase at the high W/R, while magnetites or serpentines (changeover at W/R ∽5) are the second most abundant phases at the low W/R. In contrast, a pure mineral mixture of 70% olivine, 15% augite, and 15% plagioclase returns a chlorite-serpentine assemblage with minor amounts of nontronite only—over the entire W/R range (Figure 6c), with pH varying between 10 at the higher W/R and 12 at the lower W/R. Most importantly, 70% Portage amorphous, 20% olivine, and 10% whole rock precipitates a nontronite-dominated assemblage over the entire W/R range, with pH between 10 at W/R of 10000 and 12 at W/R of 100. For this run, hematite and goethite were suppressed (Figure 6d), and the result compared to a run with hematite formation at high W/R. There are no significant differences in clay formation between the two runs. We will discuss the implications of this in a later section.

### 3.4 Variable CO_2_

**Figure 7:**
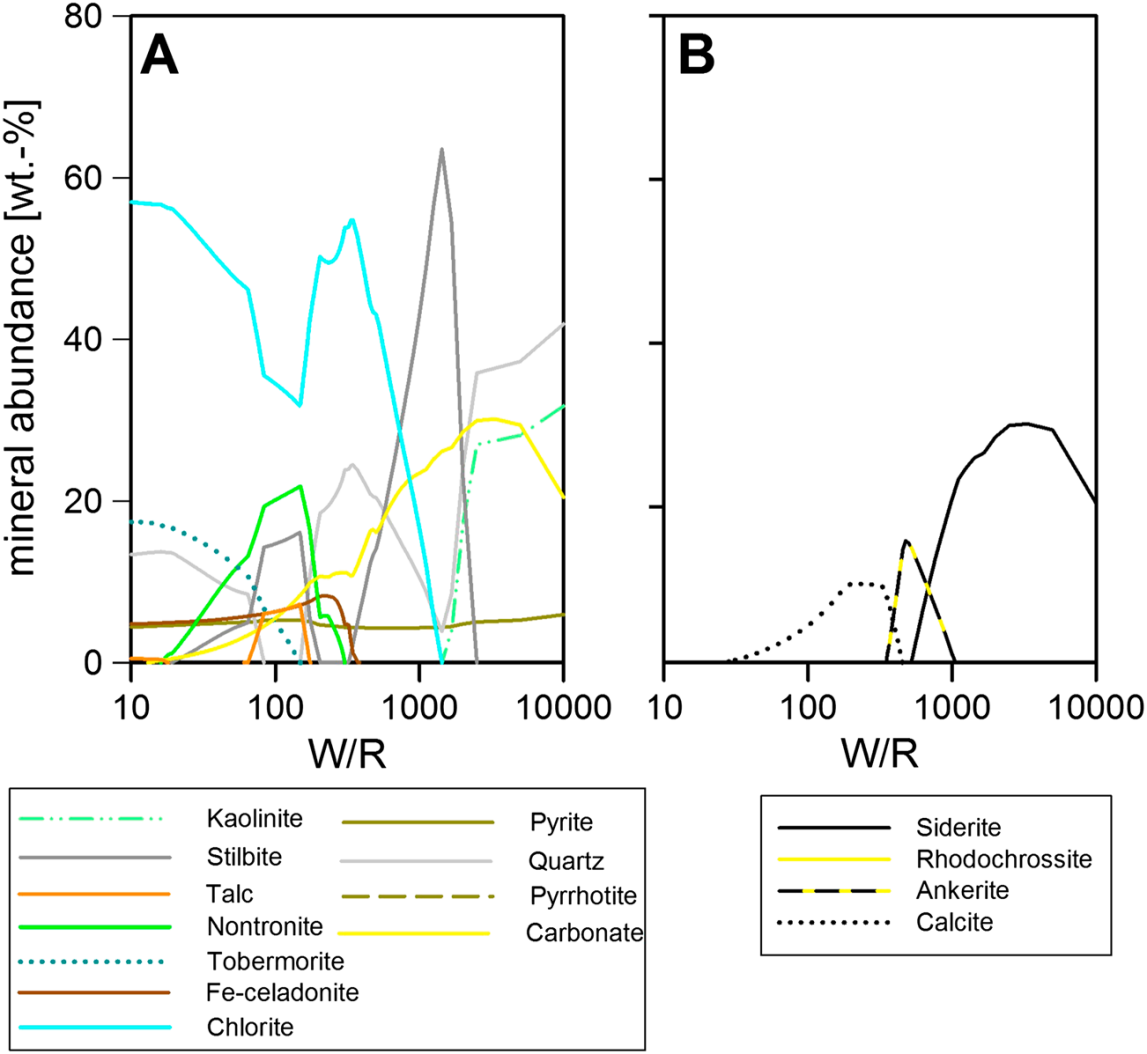
Portage soil reacted with GPW and 0.62 mol of H_2_CO_3_ added. Other parameters are 10°C, 1 bar, and 10% Fe as Fe^3+^. All models include apatite and trace phases (below 3%) are not plotted either. W/R is the ratio of incoming fluid with reacted rock.

The main differences between the systems with and without CO_2_ are observed at high W/R (Figure 7). At the highest W/R, carbonate (effectively siderite; Figure 7b) is the second most abundant phase, while the most abundant phase is an SiO_2_-phase (between W/R of 10,000 and 2000, then stilbite between W/R 2000 and 750, after which chlorite becomes the most abundant phase). In contrast to the CO_2_-poor case (composition in Figure 3a), chlorite formation is not possible, while CO_2_ concentrations are high and ion concentrations from the silicate are low, and kaolinite forms at the highest W/R (>1700). Nontronite formation, too, is not possible during the peak siderite formation (Figure 7). At intermediate W/R, more ions from the silicate dissolution become available, facilitating nontronite formation (W/R of <300). The carbonate is initially siderite (Figure 7b), between W/R of 1000 and 500 siderite-ankerite assemblage, pure ankerite in a very short interval (W/R of 500–450), then calcite-ankerite (W/R 450–330) after which the carbonate is calcite until carbonate formation ceases at W/R of 13. This example demonstrates that high HCO_3_^−^ activity would favor carbonate formation over clay [*Catalano*, 2013[, especially those that take up Fe, Mg, and Ca. Since SAM analyses allow the possibility of only minor carbonate amounts [*Ming et al*., 2014[, and CheMin found up to 22% clay, this points toward a carbonate-poor alteration scenario, within the subsurface and not able to exchange with the atmosphere, especially not a potentially thicker CO_2_-atmosphere that is generally envisaged for early Mars.

## 4. Discussion

### 4.1 Modeling of Clay-Bearing Assemblages

Modeling of low-temperature clay mineral formation on Mars has been carried out before and is always faced with a set of difficulties resulting from a lack of thermochemical data of phases likely to form. This is largely due to the extrapolation required from laboratory experiments to the relatively low temperatures in question and the level of detail at which we can actually observe the Martian alteration products. Since the modeling study of Martian clay mineral formation by *Gooding* [1978[, chlorite and serpentine assemblages have been postulated at low *T* [*Wallendahl and Treiman*, 1997[. *Chevrier et al*. [2007[ predict the pressure, temperature and composition range for nontronite formation as neutral to alkaline and under CO_2_-poor, oxidizing conditions. *Zolotov and Mironenko* [2007[ studied the alteration of a rock of shergottitic composition (EETA79001). On reacting this rock with an acidic fluid, they found the pH to increase over time. With increasing pH, the nature of the precipitated clay minerals changes from kaolinite and Fe^2+^-saponite to Mg-saponite, Na-saponite, and montmorrillonite. Modeling of low-*T*, short-lived alteration scenarios consistently predict nontronite or other clay phases after an initial acidic stage [e.g., *Hausrath et al*., 2008; *McAdam et al*., 2008; *Berger et al*., 2009[.

Another issue is to match those models to observations. *Catalano* [2013[ points out that there are no ferrous smectites in the Mars spectral databases, for which reason their orbital observation becomes uncertain. As a solution to this problem, Catalano stated that in his models Fe^2+^/Mg-saponites are predicted under reducing weathering conditions and nontronite is predicted under oxidizing weathering conditions. However, the only source of detailed, terrestrial laboratory observation of Martian clay formation are the nakhlite Martian meteorites, for which *Changela and Bridges* [2010[ and *Hicks et al*. [2014[ produced the most comprehensive analysis to date of the clay minerals in small, hydrothermal veins. The crystalline phyllosilicates are a ferric saponite and a ferric serpentine. *Bridges and Schwenzer* [2012[ used this information to model the formation conditions of the alteration succession in the nakhlite Martian meteorite Lafayette.

The alteration observed at Yellowknife Bay in many ways resembles the alteration assemblages observed in the nakhlites. This is reflected by two main observations [*Changela and Bridges*, 2010; *Hicks et al*., 2014[: in the nakhlites, (i) the alteration is of nonpervasive nature with olivine remaining within the altered nakhlites and (ii) the alteration phases precipitated are increasingly ferric throughout the course of the event. Because olivine remains in the Cumberland and John Klein samples alongside the alteration products [*Vaniman et al*., 2014[, these observations can serve as analogy between the Lafayette alteration assemblage [*Bridges and Schwenzer*, 2012[ and the Cumberland and John Klein drill samples. Thus, a moderately oxidizing host rock and an oxidizing fluid (GPW), causing a complex alteration mineral assemblages to precipitate, appear to be most likely.

### 4.2 Selective Host Rock Alteration

Pervasive alteration of the two basaltic compositions, Ekwir_brushed and Portage soil, forms a clay-rich alteration assemblage. The dominant predicted sheet silicate is chlorite, which is a consequence of the Fe^2+^ dominance in the system (and the lack of ferrous saponite in the model). This observation alone would not rule out comprehensive alteration, but the minerals accompanying the clays, e.g., SiO_2_ (at larger quantities) and zeolite, in the model (Figures 4b and 4c) are not observed by CheMin [*Vaniman et al*., 2014[. Thus, the observed alteration assemblage is not a product of pervasive alteration of the entire whole rock, and this is in line with the identification of magmatic minerals (olivine, pyroxenes, and feldspars) by CheMin.

Experiments on terrestrial basalts [e.g., *Gudbrandsson et al*., 2011[ demonstrate that olivine dissolution at pH below ∽10 is faster than dissolution of pyroxene and feldspars, and pyroxene dissolution exceeds (but to a lesser degree than olivine dissolution) feldspar dissolution between pH of ∽2 and ∽8. Thus, predominant dissolution of olivine is expected at the beginning of the mudstone alteration, when pH of the fluid is near neutral, e.g., 7.5–8.5, as occured over wide ranges of our runs.

**Figure 8:**
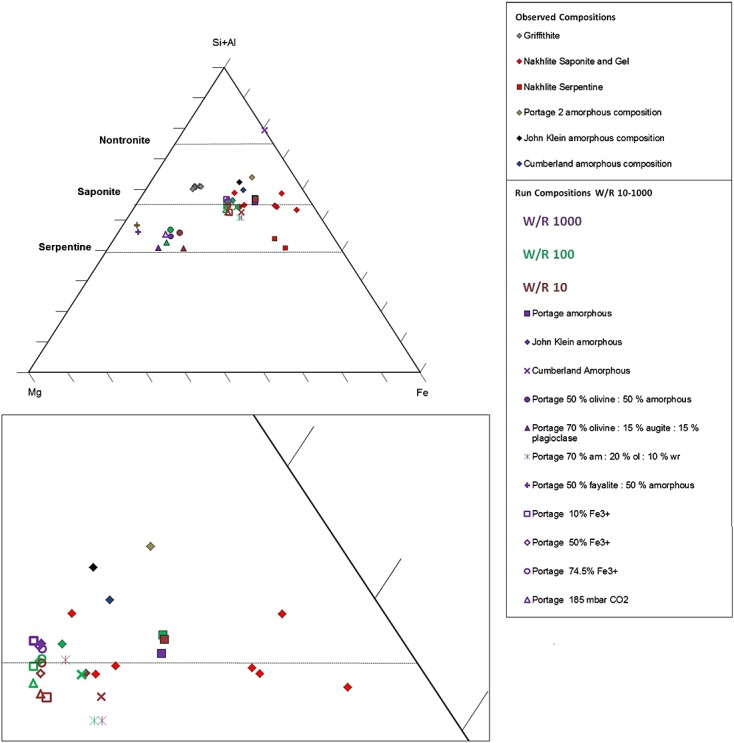
Clay Compositions on Mg-Al-Fe_tot_. The starting compositions are the Portage, Cumberland, and John_Klein amorphous compositions [*Vaniman et al*., 2014; *Morris et al*., 2014[ and the analogue comparison clays. Saponite and saponitic gel and serpentine from the nakhlites [*Hicks et al*., 2014[ and griffithite [*Treiman et al*., 2014[ are also plotted. For the run compositions, we have plotted a range of mixtures of amorphous, olivine, plagioclase, and whole rock, reacted with GPW fluid at W/R of 1000 (purple color), 100 (green color), and 10 (brown color). The Portage whole rock reacted with CO_2_-bearing GPW is also plotted, and the results of clays from runs of Portage whole rock reacted with GPW at Fe^3+^/Fe_tot_ from 10 to 75% (Figure 4). The Portage 70% amorphous, 20% olivine, and 10% whole rock reactions at W/R 1000 and 100 have compositions close to the saponite from the nakhlites. The Portage 70% olivine, 15% augite, and 15% plagioclase run at W/R 1000 and 100 are closer to Mg-rich serpentine, and this type of olivine-rich run may be analogous to the formation of Mg-rich ridges at Yellowknife Bay. W/R is the ratio of incoming fluid with reacted rock.

The other potentially reactive phase in the rocks that we considered is the amorphous component. For the Portage sample, basaltic glass or allophane are candidates for the amorphous phase [*Bish et al*., 2013[. Its chemistry in the Cumberland and John Klein drill holes suggests that it is closely related to the clay minerals [*Vaniman et al*., 2014[; potentially, it could be a poorly crystalline clay mineral component as found in the nakhlite Martian meteorites [*Changela and Bridges*, 2010; *Hicks et al*., 2014[. All three amorphous compositions produce alteration mineralogy consistent with the observed silicic alteration assemblage as determined by CheMin, but with no or minor Fe oxide phases. Because an enrichment of magnetite is observed in the mudstone compared to the Portage soil [*Bish et al*., 2013; *Vaniman et al*., 2014[ (see also section 2.2), this is considered to be part of the in situ alteration process [*Bristow et al*., 2014[, and additional Fe appears to be needed. Because olivine is observed at higher concentration in the soil compared to the mudstone, olivine dissolution is a likely source of Fe. Mixtures dominated by the amorphous component, with some added olivine and whole rock, return assemblages matching the observations best (Figures 6d and 8). We thus conclude that inhomogeneous dissolution of a rock of Portage soil composition could produce the observed alteration assemblage. Our preferred mixing ratio is 70% amorphous component, 20% olivine, and 10% whole rock. This accounts for dissolution kinetics in the complex system and returns a clay mineral dominated, Fe oxide-bearing alteration assemblage. We next compare the clay mineral chemistry to the Gale and nakhlite clays.

### 4.3 Host Rock Alteration: Clay Chemistry and Abundance

It is not possible to exactly match the clay mineralogy in our models to the reported CheMin results, so we average the composition of all sheet silicates in the system at W/R 1000, 100, and 10 (Figure 8) and compare this to the clays identified from the CheMin measurements [*Vaniman et al*., 2014[ and that found in the nakhlite Martian meteorites [*Changela and Bridges*, 2010; *Hicks et al*., 2014[ and terrestrial griffithite [*Treiman et al*., 2014[. Clay mineral compositions resulting from the reaction of Cumberland, John Klein, and Portage amorphous phases fall between the saponite and nontronite stoichiometric lines and near the most Mg-rich compositions found in the Lafayette meteorite. In contrast, if olivine alteration dominates the alteration assemblage (mixtures with 50% and 70% olivine; Figure 6), the resulting clay composition is serpentine-rich and falls well outside the nakhlite field or griffithite, which are analogues for the saponite determined by CheMin (Figure 8). As argued in the previous section, olivine is needed to match the Fe oxides in the assemblage. The preferred approximate mix of 70% amorphous component with 20% olivine and 10% host rock dissolution produces a saponite similar in Si + Al concentration to the amorphous component alone. In summary, our clay from the 70:20:10 mix has—based on (Si,Al)_4_—a saponitic composition of (Na,K)_0.08_Ca_0.38_ Mg_1.10_Fe_1.54_(Si,Al)_4_O_10_(OH)_2_ at W/R of 100 and (Na,K)_0.17_Ca_0.38_ Mg_1.08_Fe_1.33_(Si,Al)_4_O_10_(OH)_2_ at W/R of 10.

However, if the olivine preferentially reacts before the other phases and thus dominates the alteration assemblage at an early stage then this is a possible explanation for the Mg-rich ridges described by Leveille et al. [2014[. On Figure 8, we have plotted the Portage 70% olivine, 15% augite, and 15% plagioclase; 50% forsterite and 50% Portage amorphous; and the 50% fayalite and 50% Portage amorphous runs at different W/R. They plot closer to the Mg corner than the other runs and close to serpentine. This possible explanation for the Mg-rich ridges is considered further in section 4.5.

The presence of ∽20% clay in the Sheepbed mudstone is consistent with our model. The rock mixture dissolves, so 100% of the silica in the alteration assemblage stems from the rock dissolving in situ. For instance, at W/R of 1000 (with our 70% amorphous/20% olivine/10% bulk model, GPWox dilute starting fluid), we have 0.64E-02 mol of Si in the system, of which 0.11E-02 mole are in the resultant fluid. This means that a maximum of 20% of the silica is in the fluid after the reaction and could potentially have left the system. Silica from the “fluid in” component is negligible; it is in the 10E–5 range, 3 orders of magnitude smaller. If we look at silica at W/R of 100, then we predict 0.65E-01 mol in the system (again, the contribution from the incoming water is very small) and 0.37E-02 mol in the fluid. So there is now 6% remaining in the fluid. This means that for silica, between 80 and 94% of the silica from the rock can be deposited as clay. In other words, at W/R of 1000, the redeposition is almost complete and 20% clay alteration product would stem from dissolving the preexisting rock. Therefore a W/R ratio of 100–1000 is consistent with the magnitude of clay abundances in the Sheepbed mudstone.

### 4.4 Fe Oxides and Fe^2+^/Fe^3+^ Ratios in the Clay Minerals

The amount and nature of the Fe oxide precipitated at low to intermediate W/R is dependent on a complex interplay of the overall redox conditions of the system, the assemblage of Fe-bearing phases and their redox states. For nontronite (or ferric saponite) formation, the fluid and the host rock need to provide Fe^3+^ or conditions to oxidize iron. We note that in the models shown in this study, the fluid at the start of the alteration is oxidizing (see section 2.1). This is in accordance with the observed presence of oxychlorine species in the sediment [*Ming et al*., 2014[.

Modeling of pure olivine alteration was carried out by *Bristow et al*. [2014[, and this did not return significant amounts of magnetite at the temperature range considered here for diagenesis (10–50°C), but—similar to our observations in high-*T* runs (not shown)—the amount of magnetite precipitated increases at higher temperatures. At this point, it is important to bear in mind that post formational processes can change the oxidation state of Fe [*Cornell and Schwertmann*, 2003; *Leveille et al*., 2014[, even if the Yellowknife Bay rocks were never heated above 60°C (see *Bristow et al*. [2014[ for a more in-depth discussion of the magnetite topic). CheMin observation suggests a partial oxidation of the ferrous iron in magnetite [*Vaniman et al*., 2014[. As expected, magnetite formation (in contrast to pure Fe^3+^-oxide formation) appears to correlate with the absence or decrease in ferric relative to ferrous clay (see Figures 5b and 6b).

Comparing modeled to observed Fe^3+^/Fe_tot_ ratios, using the relative proportions of nontronite and ferrous chlorite, reveals the comparatively ferrous nature of the Yellowknife Bay clay: the modeled ratio varies between 0.3 and 0.5. This is fairly low compared to the terrestrial griffithite proposed and used as an analogue for the Curiosity clay mineralogy [*Treiman et al*., 2014[, which has Fe^3+^/Fe_tot_ ratios of 0.6 to 0.9. The nakhlite saponite and amorphous gel of saponite composition have Fe^3+^/Fe_tot_ ratios of 0.3–1.0 [*Hicks et al*., 2014[. The relatively ferrous nature of the saponite supports our model predictions of magnetite over hematite formation alongside the clay.

### 4.5 Sequence of Events

As shown above, the dissolution dominated by a mixture of amorphous phase and olivine can explain the major clay forming event; it cannot, however, account for the entirety of alteration features observed in the geologic sequence of events at Yellowknife Bay. We will next expand our discussion to link into the diagenetic sequence described at Yellowknife Bay by *Grotzinger et al*. [2014[: deposition of the lake bed sediments, followed by early diagenetic formation of features called “raised ridges” and nodules, followed by the main clay forming event, followed by the formation of late sulfate veins.

#### 4.5.1 Sedimentation and Early Diagenetic Features

After the deposition of the lake bed sediments (most of which are fine, medium, and coarse grained sandstones [*Grotzinger et al*., 2014[), the first diagenetic features observed to form are raised ridges, nodules, and minibowls (hollows). All models of nodule formation agree on their early diagenetic nature, and hypothesis of formation are gas bubbles [*Leveille et al*., 2014; *Siebach et al*., 2014; *Stack et al*., 2014[. We note that some of the reactions in our models, for example, the reaction of Ekwir with GPW at 50°C, would produce a water vapor dominated gas phase. However, with olivine still remaining in the host rock, gases from local water-rock reactions are probably not an important contribution from the reactions in the Yellowknife Bay sediment, and so we do not consider them in detail in this paper.

The raised ridges are more resistant to weathering than the surrounding sediment and show elevated Mg, Fe, Si, and Cl concentrations [*Grotzinger et al*., 2014; *McLennan et al*., 2014; *Leveille et al*., 2014[. The proposed formation scenarios include subaerial and subaqueous settings. From our models, we can exclude subaerial contact of the fluid with the CO_2_-rich Martian atmosphere during precipitation of the minerals, because the availability of CO_2_ in the system would cause carbonate precipitation but also the formation of zeolites, quartz/SiO_2_, and kaolinite (Figure 7), none of which are observed at the relevant quantities in the CheMin data. Higher Mg and Fe activities in the incoming fluid or selective dissolution of phases are required in order to produce the Mg-rich phyllosilicate assumed to be the cementing material of the raised ridges. Moreover, the high concentrations of Mg (up to 17 wt % in individual ChemCam spots) found in the raised ridges [*Leveille et al*., 2014; *McLennan et al*., 2014[ with no carbonate preclude contact with the Martian atmosphere. If the high-Mg phase is 20% of the McGrath raised ridge rock, *Leveille et al*. [2014[ deduced a potential composition of the alteration phase of 45 wt % SiO_2_, 35 wt % FeO, and 35 wt % MgO.

In accordance with the observations in Icelandic low-temperature surface fluids [*Gíslason and Arnórsson*, 1993; *Stefánsson et al*., 2001[, forsteritic olivine could preferentially dissolve over fayalitic olivine, especially on first contact with a fluid or under oxidizing conditions [*Hausrath and Brantley*, 2010[. If we assume that during the early diagenetic phase, the olivine component and amorphous phase initially dissolved at a faster rate than the other phases, then a clay alteration phase from our runs with ∽50% SiO_2_ and ∽40% MgO, and ∽5% FeO (plus Al_2_O_3_, CaO, Na_2_O, and K_2_O) forms alongside ∽10% Fe oxide/hydroxide. The assemblage is slightly dependent on W/R but overall similar at all W/R as shown by our Portage, 50% fayalite or forsterite and 50% amorphous runs plotted on Figure 6 and 8.

#### 4.5.2 Main Diagenetic Phase: Clay Formation

The second stage is the main clay forming event—as discussed above—and occurs by the reaction of the local pore fluid (GPW) and selective dissolution of the basaltic sediments, in detail, a mixture of 70% amorphous material with 20% olivine and 10% host rock (all compositions from Portage soil). This stage is pervasive, and olivine dissolution is congruent. This calculation results in a good match of the modeled clays to the CheMin observations—and in fact the nakhlite clays, too. We assume the W/R of the reaction is higher than 10, because at very low W/R talc formation exceeds 10%, but talc is not observed in the CheMin data. However, the bulk W/R, given the preservation of olivine in the rocks, is likely not high. The pH at W/R of 1000 to 10 changes from around 7.5 to 12 as the alteration progresses from that associated with the Mg-rich ridges and initial olivine dissolution to the main diagenetic assemblage of saponite and magnetite.

After this main clay-forming event, the sulfate veins formed [*Grotzinger et al*., 2014; *Nachon et al*., 2014[. Their formation requires enrichment of dissolved species—by freezing or evaporation—in fractures of the rock. Because this work focuses on the clay formation, detailed modeling of the last stage is beyond the scope of this work and discussed elsewhere [*Schwenzer et al*., 2014, and in preparation[.

#### 4.5.3 CO_2_ and the Alteration Assemblage

While orbital observations have suggested ice, permafrost, and liquid water during the earliest history of Gale Crater [e.g., *Cabrol et al*., 1999; *Pelkey and Jakorsky*, 2002; *Pelkey et al*., 2004; *Thomson et al*., 2011; *Schwenzer et al*., 2012a; *Fairén et al*., 2014, and references therein[, with Curiosity's observation of conglomerates [*Williams et al*., 2013[, mudstones [*Grotzinger et al*., 2014[, and clay minerals [*Vaniman et al*., 2014[ (Table 1), it is clear that there was liquid water on the surface and in the subsurface after the formation of Gale Crater, which suggests conditions under which pressure and temperature combinations reached the water stability field for at least part of the year. From our models, we can exclude contact of the fluid with the CO_2_-rich Martian atmosphere during precipitation of the minerals, because the availability of CO_2_ in the system would cause not only significant carbonate precipitation but also the formation of zeolites, quartz/SiO_2_, and kaolinite (Figure 7), none of which are observed at the relevant quantities in the CheMin data.

## 5. Conclusions

1. The reaction of sedimentary material of basaltic composition, similar to Portage soil, with a pore fluid can explain the main mineralogical features of the diagenetic assemblage identified in Yellowknife Bay, Gale Crater.
2. We have tested the reaction of more alkaline compositions identified in Gale Crater and hydrothermal temperatures at 150°C. Neither scenario can account for the reported mineral assemblages.
3. The fluid (we call it Gale Portage Water (GPW)) is a dilute aqueous solution derived from the mediation of a brine with the cation and anion contents in equilibrium with rocks of the Gale area.
4. An early stage of diagenesis associated with Mg-rich ridges suggest some initial, localized alteration reactions associated with the early preferential alteration of olivine with GPW.
5. Inhomogeneous host rock dissolution of predominantly amorphous phase identified by CheMin, with lesser olivine and minor overall host rock contribution, occurred via reaction with GPW at 10–50°C, with a W/R of 100–1000, and pH of 7.5–12. This occurred in an open system with fluid flow and led to a clay-Fe oxide assemblage. The bulk compositions of the modeled phyllosilicate assemblages are similar to saponite clays observed at Yellowknife Bay and in the Lafayette Martian meteorite, though more ferrous with Fe^3+^/Fe_tot_ ratio of 0.3–0.5. The resultant relatively ferrous phyllosilicate produced is consistent with the dominance of magnetite rather than ferric oxide at a W/R of 100 and below. However, only a minor change in the redox state of the fluid might trigger magnetite formation at higher W/R.
6. The reactions associated with the clay and magnetite formation did not occur in a setting where exchange with an overlying CO_2_-rich atmosphere was possible. This is predicted by the absence of a significant carbonate abundance and the absence of phases including zeolites that our models predict are likely to be associated with such a CO_2_-charged fluid.

## Key Points

- Thermochemical modelling constrains the secondary minerals in Gale Crater
- Inhomogeneous dissolution of amorphous and olivine phases forms clay, magnetite
- Formed by reaction of CO_2_-poor, oxidising brine with basaltic sedimentary rocks
